# Video Q & A: The impact of stress. An interview with George Chrousos

**DOI:** 10.1186/1741-7015-12-102

**Published:** 2014-06-20

**Authors:** George Chrousos

**Affiliations:** 1 Division of Endocrinology, Metabolism and Diabetes, University of Athens Medical School, Aghia Sophia Children's Hospital, Athens 115 27, Greece; 2 National Institute of Child Health and Human Development (NICHD), National Institutes of Health (NIH) Bethesda, Maryland 20892, USA

## Abstract

In this video Q & A, we talk to George Chrousos about stress and its impact on chronic non-communicable disorders (NCDs) and early life development. Professor Chrousos also discusses the effects of psychological and economical stressors on health, and suggests ways in which we can learn to cope with stress.

## An interview with George Chrousos

### Introduction

George Chrousos is currently the Chairman of the First Department of Pediatrics at the University of Athens School of Medicine, Greece and the former Chief of the Pediatric and Reproductive Endocrinology Branch of the National Institute of Child Health and Human Development (NICHD), National Institutes of Health (NIH), USA. He also holds the UNESCO Chair on Adolescent Health Care at the University of Athens and held the 2011 John Kluge Distinguished Chair in Technology and Society at the Library of Congress, Washington, D.C. His research has focused on the glucocorticoid signaling system, diseases of the hypothalamic-pituitary-adrenal (HPA) axis and on the physiological and molecular mechanisms of stress. His research contributions span a wide range of medical disciplines, and his early ground-breaking research first described the syndrome Primary Generalized Glucocorticoid Resistance (more recently termed “Chrousos syndrome”), which is a rare genetic or sporadic condition characterized by glucocorticoid insensitivity. Professor Chrousos’s recent research includes the management of stress during pregnancy and the genetic variation of the HPA in relation to the stress response in asthmatic children.

## Edited transcript

### (1) How would you differentiate between stress and stressors?

First, we need to discuss homeostasis, which is basically the balance or the equilibrium that human beings or living organisms need to maintain. Stressors are the stimuli that disturb or threaten homeostasis. The adaptive response is what the organism is doing to antagonize the stressor, and stress is the state of threatened homeostasis, so when an organism is under stress then there is a stressor that disturbs that organism’s homoeostasis.

### (2) What biological systems are associated with the stress response?

During the stress response the entire organism participates; however, there is a system which is called the ‘stress system’ that is activated once the stressor exceeds a certain threshold. This system consists of brain and peripheral components. In the brain the paraventricular nucleus in the hypothalamus contains neurons that secrete corticotropin- releasing hormone, which then circulates in the hypophysial portal system to the pituitary where it stimulates the secretion of adrenocorticotropic hormone (ACTH). In the systemic circulation ACTH stimulates the secretion of cortisol from the adrenal cortex. In addition, there is another center in the brain called the locus caeruleus/norepinephrine system, which is in the brainstem and is the center of the arousal and autonomic nervous systems which consist of the adrenomedullary and the systemic sympathetic system components.

### (3) What disorders are affected by the stress system?

The stress system when activated can precipitate acute and chronic disorders. For acute disorders, for example, a stressor can precipitate an asthma attack, a hypertensive episode or a psychotic episode. For chronic conditions, this is more serious, as chronic stress can result in several chronic non-communicable diseases, including obesity, metabolic syndrome, diabetes mellitus type 2, hypertension, depression and anxiety.

### (4) What is the role of genetic vulnerability to stressors in our environment?

Our vulnerability to stressors depends to a great extent on our genes (polygenic) and also on our epigenetics, which express the effect of our environment on our genetic material.

### (5) What are the long-term effects of stress in prenatal and early life?

Prenatal and early life, (especially the first five years of life), are extremely vulnerable periods and that is why they are called critical or critically vulnerable periods. If there is stress during this time the stress hormones leave behind an epigenetic imprint, which accompanies the child for the rest of his or her life into adulthood and old age.

### (6) How do psychological and economic factors affect stress?

In modern society most of the stress we are exposed to is social and, in a sense, to a large extent, anthropogenic. The economy is part of social life, so if someone does not have the economic means to live a normal life and needs to scrounge around for resources, then this causes stress which is damaging to the organism. There is no distinction between financial, social and peer-generated stress and they are all damaging through the same pathophysiologic mechanisms.

### (7) What are the proven methods that prevent stress?

Two things need to be done when faced with stress; first, one has to eliminate the stressor or get away from it, which is a smart move; for example, if someone is in a stressful job then that person needs to consider changing jobs. However, if this cannot be done, then that individual needs to learn to cope with stress and there are many ways to do this. These include leading a healthy life, which involves (1) eating a good diet and high quality nutrition; (2) performing exercise regularly; (3) having good timing of daily activities, for example, waking up and having breakfast should be at approximately the same time of the day and (4) sleeping well. Unfortunately, in the last 30 years we sleep on average two hours less than the duration of sleep of people in the 1970s and this is stressful for the body as it alters metabolism. It is very well known now that the lack of sleep ultimately damages the blood vessels and leads to chronic stress in an individual.

### (8) What are the future directions of this important topic of research?

First, the message needs to be promoted. Second, there is a requirement for social and political changes that will guarantee that our children are not exposed to stress but have enriching experiences. Third, society needs to learn how to cope with situations that cannot be avoided. So far it seems that one of the psychiatric and psychological methods that is used quite successfully to cope with chronic social stress is cognitive behavioral therapy.

### (9) Where can I find out more?

See references [1-13].

## Competing interests

GC has no competing interests to declare.

## Supplementary Material

An interview with George Chrousos Click here for file

## References

[B1] Chrousos GP Primary generalized glucocorticoid resistance BMC Med 2011 9 27 10.1186/1741-7015-9-27 21429213 PMC3086527

[B2] Chrousos GP Gold PW The concepts of stress and stress system disorders: overview of physical and behavioral homeostasis JAMA 1992 267 1244 1252 10.1001/jama.1992.03480090092034 1538563

[B3] Chrousos GP The hypothalamic-pituitary-adrenal axis and immune-mediated inflammation N Engl J Med 1995 332 1351 1362 10.1056/NEJM199505183322008 7715646

[B4] Elenkov IJ Chrousos GP Stress hormones, Th1/Th2-patterns, pro/anti- inflammatory cytokines and susceptibility to disease Trends Endocrinol Metab 1999 10 359 368 10.1016/S1043-2760(99)00188-5 10511695

[B5] Makrigiannakis A Zoumakis E Kalantaridou S Coutifaris C Coukos G Rice K Gravanis A Chrousos GP Corticotropin-releasing hormone (CRH) promotes blastocyst implantation and early maternal tolerance Nat Immunol 2001 2 1018 1024 10.1038/ni719 11590404

[B6] Pervanidou P Kolaitis G Charitaki S Lazaropoulou C Papassotiriou I Hindmarsh P Bakoula C Tsiantis J Chrousos GP The natural history of neuroendocrine changes in pediatric post-traumatic stress disorder (PTSD) after motor vehicle accidents: progressive divergence of noradrenaline and cortisol concentrations over time Biol Psychiatry 2007 62 1095 1102 10.1016/j.biopsych.2007.02.008 17624319

[B7] Chrousos GP Stress and disorders of the stress system Nat Rev Endocrinol 2009 5 374 381 10.1038/nrendo.2009.106 19488073

[B8] Chrousos GP Stress and sex *vs*. immunity and inflammation Sci Signal 2010 3 pe36 20940425 10.1126/scisignal.3143pe36

[B9] Charmandari E Chrousos GP Lambrou GI Pavlaki A Koide H Ng SS Kino T Peripheral CLOCK regulates target-tissue glucocorticoid receptor transcriptional activity in a circadian fashion in man PLoS ONE 2011 6 e25612 10.1371/journal.pone.0025612 21980503 PMC3182238

[B10] Pervanidou P Chrousos GP Posttraumatic stress disorder in children and adolescents: neuroendocrine perspectives Sci Signal 2012 5 pt6 23047921 10.1126/scisignal.2003327

[B11] Charmandari E Achermann JC Carel JC Soder O Chrousos GP Stress response and child health Sci Signal 2012 5 mr1 23112343 10.1126/scisignal.2003595

[B12] Kino T Charmandari E Chrousos GP Fink G, Pfaff DW, Levine JE Chapter 29. Disorders of the hypothalamic-pituitary-adrenocortical system Handbook of Neuroendocrinology 2012 London, UK: Waltham, MA; San Diego, CA: Academic Press, Elsevier 639 658

[B13] Chrousos GP Stress in early life: a developmental and evolutionary perspective Z. Hochberg Evo-Devo of Child Growth 2012 Hoboken, NJ: Wiley-Blackwell Co 29 40

